# Comparing Coronal Discoloration Between AH26 and ZOE Sealers

**Published:** 2011-11-15

**Authors:** Maryam Zare Jahromi, Amir Arsalan Navabi, Mahsa Ekhtiari

**Affiliations:** 1. Department of Endodontics, Dental School, Islamic Azad University of Medical Sciences, Khorasgan Branch, Isfahan, Iran.; 2. Islamic Azad University of Medical Sciences, Khorasgan Branch, Isfahan, Iran.; 3. General Dentist, Isfahan, Iran.

**Keywords:** AH26 Sealer, Tooth Discoloration, Zinc Oxide Eugenol

## Abstract

**INTRODUCTION:**

Intrinsic tooth discolorations after endodontic treatment are principally attributed to the composition of necrotic pulp tissue, hemorrhage within the pulp cavity, endodontic medicaments and/or filling materials. Residual sealer left in pulp chamber after obturation can cause discoloration. The objective of this in vitro study was to evaluate coronal discoloration created by AH26 and ZOE sealers after four months.

**MATERIALS AND METHODS:**

Fifty intact human extracted maxillary central incisors were employed. Access cavities were prepared in all samples and root canals were instrumented; coronal orifices were then sealed using self-cure glass ionomer. The teeth were divided into two experimental groups (n=20) according to utilized sealer in pulp chambers including AH26 and Dorifill (ZOE). The remaining 10 teeth served as negative and positive controls (n=5). The access cavities were sealed with self-cure glass ionomer. Teeth were kept in incubator for four month. Preliminary digital images of the teeth were taken and then compared with those related to 4-month follow-up. The images were assessed using Photoshop software. Data was analyzed using paired t-test and independent samples t-test.

**RESULTS:**

The teeth which were filled with AH26 sealer showed significantly greater discoloration than those filled with ZOE sealer (Dorifill) (P<0.05).

**CONCLUSION:**

AH26 sealer causes greater discoloration of the crown compared to ZOE sealer. Despite the other disadvantage of AH26 sealer, it seems that Dorifill is more esthetically considerate.

## INTRODUCTION

Anterior tooth discoloration is an esthetic problem for both the patient and dentist. Sources of coronal tooth discoloration can be natural (acquired) or iatrogenic (inflicted). Natural causes are those that occur as a result of tooth developing disturbances or due to patients’ behavior, tooth caries, or traumatic injuries [1][2][3][4].

Iatrogenically induced discoloration results from different factors such as restorative or obturating materials. Many of the materials that are used in dentistry have the potential to cause discoloration i.e. amalgam, Cavit, IRM, some drugs such as chlorhexidine, fluoride and intracanal medicaments such as phenolics and iodoform-based medicaments [5][6][7].

The most common cause of coronal discoloration is presence of remnant sealer in the pulp chamber that causes sealer to infiltrate into dentine tubules leading to discoloration [8]. If the materials are not removed from the pulp chamber after obturation, subsequent staining may occur [9].

On the basis of results of literature most discolorations occur at the midcervical surface of teeth where enamel is thin and enamel translucency makes dentinal discoloration apparent [10]. Scanning electron microscopies have shown that the presence of the smear layer obstructs the penetration of sealer into the dentinal tubules [11][12][13].

Sealer infiltration and discoloration is related to some factors e.g. dentine thickness and sealer quality. Therefore dentists require adequate knowledge of sealers’ properties. Davis et al. examined coronal discoloration of seal apex sealers, Roth 801 (Kerr, Romulus, MI., USA) and AH26 (Detrey, Dentsply, Germany). They found that all sealers cause coronal discoloration within few weeks and AH26 created the greatest discoloration [10]. Another study examined coronal discoloration of four sealers including AH26, Roth 801, Kerr sealer and Sealapex; they found that even silver free sealers cause coronal discoloration. AH26 was reported to have the greatest discoloration effect [14].

The aim of this in vitro study was to compare discoloration caused by AH26 and ZOE sealers after four months.

## MATERIALS AND METHODS

Fifty intact human maxillary central incisors were included in this study; inclusion criteria were teeth with no caries, restorations, developmental defect, and coronal discoloration. The teeth were cleaned with ultrasonic to remove gross debris, followed by rubber cup and pumice to remove remaining debris and to remove stains from the coronal crown surface.

**Figure 1:**
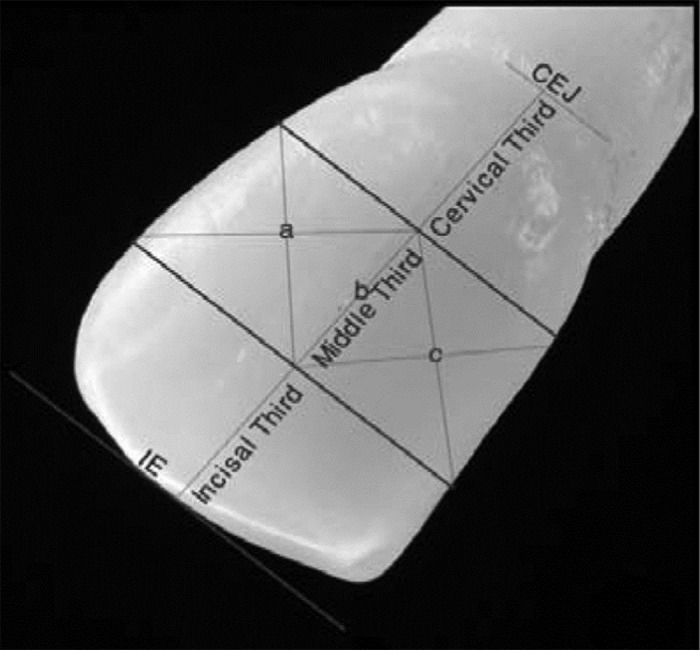
The Labial surface of tooth (Three points a, b and c)

For evaluating color, digital photography was taken under the same brightness, light source and environment on the black sheet. Labial surface were photographed and pictures were transferred to a computer (Figure 1). RGB (Red, Green and Blue colors) and HSB (Hue, Saturation and Brightness) variables used as a criterion to determine color in three points of labial surface of samples (Figure 1) using Photoshop software.

Then access cavities were prepared in all samples. Gates-Glidden drills no. #1, 2, 3 (Mani, Tochigi, Japan) were used for coronal enlargement and canals were prepared manually with K-files Mani, Utsunomiya, Japan) in step-back technique up to size 25 as master apical file. Canals were dried using paper cones (Ariadent, Tehran, Iran) and then coronal orifices were sealed 1 to 2mm using self-cure glass ionomer.

Teeth were randomly assigned to experimental and control groups. Forty teeth were divided into two experimental groups of 20 teeth each and 10 teeth were used as positive and negative controls (n=5). In the group A, pulp chamber was filled with AH26, and in group B pulp chambers received Dorifill sealer. We used surgical curette for placing sealers. After all the sealers set (while samples were kept in wet gauzes), access cavities were sealed by self cure glass ionomer (Chemfil II, De Trey Dentsply, Konstanz, Germany). In the negative control group, above process except sealer administration was performed and the positive controls were stained internally by filling the chamber with lysed red blood cells [15].

After assurance of glass ionomer setting and complete cavity seal, samples were put in normal saline and kept in incubator for 4 months at 37°C; every three days normal saline solution was changed. After 4 months images were taken from samples with the same angulations, magnification and condition. Photographs were again transferred to a computer and the same three points on labial surface as previous measurement were evaluated for HSB and RGB variables by Photoshop software. The HSB and RGB variables of each sample were determined. Data was analyzed using paired t-test and independent samples t-test.

## RESULTS

The difference in each variable of HSB before and after using sealer was analyzed using paired samples t-test. Results showed that mean discoloration of AH26 sealer (Group A) in each parameter (Hue, Saturation and Brightness) was greater than Dorifill (Group B) (P<0.05) using independent samples t-test. Mean differences in each parameter (Red, Green, Blue) for AH26 was higher composed to Dorifill; this difference was significant (P<0.05)(Table 1).

**Table 1:** The Mean±SD of discoloration Of HBS and RGB in different groups

**Groups**	**R**	**G**	**B**	**H**	**B**	**S**
**Dorifill(ZOE) **	-1.7±2.3	-3.6±1.2	-3.3±2.5	0.0015±2.8	-2.5±4.8	-1.5±1.8
**AH26**	4.6±4.1	-11.6±9.4	18.6±7.3	1.8±3.9	1.9±3.5	-8.4±3.2
**Positive control**	58.6±15.3	-52±13.1	-42.4±12.4	1.4±2.7	22.9±8.4	7.5±4.1
**Negative control**	-6.6±5.2	-0.1±1.4	-0.7±1.2	3.1±4.1	-2.7±3.3	-2.1±3.4

## DISCUSSION

Ideal sealer showed have characteristics such as good adhesion, adequate seal, radiopacity, dimensional stability during setting, tissue tolerance, antibacterial effect, non-resolvability in tissue fluids and cause no discoloration for the tooth structure [3]. The most common cause of coronal discoloration is presence of remaining sealer in pulp chamber which causes sealer to infiltrate into dentine tubules leading to discoloration [8]. Most sealers show some degrees of tooth discoloration. AH26 and Dorifill tooth discolorations were compared in this in vitro study. HSB (H=Hue), (S=Saturation) and (B=Brightness or value) and RGB variables were measure as a base for evaluating samples colors. This method seems to be more sensitive in detecting color changes than clinically visual method that Parsons et al. used in their study [16]. The term, hue, refers to the actual color (i.e. red), whereas saturation is a measure of chroma or color intensity (i.e. cherry red versus brick red). Brightness describes shades of gray within a particular hue (i.e. pink versus red).

In Van der Burgt et al., Parsons et al. and our study, application of sealer into the pulp chambers was similar [8][16]. Van der Burgt et al. used EDTA and NaOCl for irrigation during canal preparation, whereas Parsons et al. used only NaOCl [8][16]. In a clinical situation, effort should be made to remove as much of the sealer as possible. Remnants of sealer, however, are often left behind, which are likely will induce some discoloration.

Results obtained from this study concurred with Van der Burgt et al. results; they showed that AH26 sealers cause a grayish discoloration and ZOE sealers cause a light red to orange discoloration [8]. In this study, AH26 showed more grayish discoloration according to the brightness parameter of HSB. In Kraus and Jordan study which inspected sealer infiltration into dentine tubules, presence of sealer in the pulp chamber was the recognized cause of discoloration and also AH26 known to have the most discoloration effect, which agrees with results obtained from the current study [14]. Parsons et al. study also confirmed that AH26 causes the greatest discoloration [16].

## CONCLUSION

In this in vitro study, AH26 causes greater mean discoloration compared to Dorifill sealer after 4 months. Therefore, Dorifill (ZOE) sealers seem more appropriate for root canal treatment of anterior teeth. Similar studies with more samples and type of sealers and also periodical discoloration assessments are recommended.
